# A miRNA-based signature predicts development of disease recurrence in HER2 positive breast cancer after adjuvant trastuzumab-based treatment

**DOI:** 10.1038/srep33825

**Published:** 2016-09-21

**Authors:** F. Du, P. Yuan, Z. T. Zhao, Z. Yang, T. Wang, J. D. Zhao, Y. Luo, F. Ma, J. Y. Wang, Y. Fan, R. G. Cai, P. Zhang, Q. Li, Y. M. Song, B. H. Xu

**Affiliations:** 1 Department of Medical Oncology, Cancer Hospital, Peking Union Medical College and Chinese Academy of Medical Sciences, National Cancer Center, Beijing, China; 2 The VIPII Gastrointestinal Cancer Division of Medical Department, Peking University Cancer Hospital and Institute, 52 Fucheng Road, Haidian District, Beijing, China; 3 State Key Laboratory of Molecular Oncology, Cancer Hospital, Chinese Academy of Medical Sciences and Peking Union Medical College, Beijing, China; 4 Department of Cancer Epidemiology, Cancer Hospital, Chinese Academy of Medical Sciences and Peking Union Medical College, Beijing, China; 5 Tumor Marker Research Center, Cancer Institute and Hospital, Chinese Academy of Medical Sciences and Peking Union Medical College, Beijing, China

## Abstract

Approximately 20% of HER2 positive breast cancer develops disease recurrence after adjuvant trastuzumab treatment. This study aimed to develop a molecular prognostic model that can reliably stratify patients by risk of developing disease recurrence. Using miRNA microarrays, nine miRNAs that differentially expressed between the recurrent and non-recurrent patients were identified. Then, we validated the expression of these miRNAs using qRT-PCR in training set (n = 101), and generated a 2-miRNA (miR-4734 and miR-150-5p) based prognostic signature. The prognostic accuracy of this classifier was further confirmed in an internal testing set (n = 57), and an external independent testing set (n = 53). Besides, by comparing the ROC curves, we found the incorporation of this miRNA based classifier into TNM stage could improve the prognostic performance of TNM system. The results indicated the 2-miRNA based signature was a reliable prognostic biomarker for patients with HER2 positive breast cancer.

Breast cancer is the leading cause of cancer-related death in women worldwide^1^, and approximately a quarter of breast cancer present with HER2 gene amplification^2^,^3^,^4^. Trastuzumab, which targets the HER2 protein and thus inactivates the downstream signal pathway, has been demonstrated to significantly reduce the possibility of tumor relapse by 50% in patients with HER2 positive breast cancer after complete resection^5^,^6^,^7^. However, despite this remarkable efficacy, there are still 15–22% of patients developing tumor recurrence or metastasis after receiving adjuvant trastuzuamb, most of which are incurable and fatal. This represents a key challenge and thus a robust prognostic model that can effectively identify patients with poorer outcome is urgently required.

Limited prognostic information is provided by traditional clinical factors. Recent studies reported that higher T stage, lack of hormonal receptor (HR) expression may be useful to assess the risk of disease development for patients receiving adjuvant trastuzumab^8^. On the other hand, abundant tumor-infiltrating lymphocytes and the absence of lymphovascular invasion were found to be favorable, independent prognostic factors for disease free survival (DFS) in patients with HR−/HER2+ breast cancer^9^. Besides, in the HR+/HER2+ subgroup, the regional heterogeneity affected DFS^10^. Nevertheless, the robustness of those clinicpathological factors is inadequately examined. Therefore there is still a need to improve the prognostic value of the current staging system, which may be accomplished by the use of molecular biomarkers.

Several studies investigate the molecular predictors of patients with HER2 positive breast cancer. It was reported that single nucleotide polymorphism (SNP) of metastasis-associated in colon cancer-1 (MACC1) gene, a key regulator of the HGF/MET pathway, was significantly associated with clinical outcome in HER2 positive breast cancer. Increased risk for progression or death was observed in carriers of the G-allele of rs1990172 and T-allele of rs975263, respectively. While C-allele of rs3735615 showed a significant protective impact on event-free survival as well as overall survival^11^. In another study, the presence of HER2/HER3 heterodimers and the loss of p21 expression were also discovered to predict a significantly poorer clinical outcome in patients when submitted to adjuvant chemotherapy and trastuzumab. But these biomarkers still require validation and are not part of standard clinical practice.

miRNAs are evolutionally conserved, small (18–25 nucleotides), endogenously expressed RNAs, which has emerged as critical modulators involved in malignant activity^12^. Furthermore, several miRNAs have been reported to be aberrantly expressed in HER2 positive breast cancer cell lines and associated with the resistance to anti-HER2 treatment^13^,^14^,^15^,^16^,^17^. However, the prognostic value of miRNA expression in clinical tumor tissue is not fully tested in this specific population.

In this study, patients with HER2 positive breast cancer who had undergone radical resection and completed adjuvant chemotherapy and trastuzumab were enrolled. We performed the comprehensive miRNA analysis and generated a multi-miRNA based signature to predict DFS. Prognostic accuracy of this classifier was assessed in training set and internal testing set, and further confirmed in an independent testing set. We also compared the prognostic efficacy with traditional clinical factors.

## Results

### Development of the miRNA prognostic classifier

A total of 211 patients who have undergone radical surgical resection with histologically negative resection margins followed by adjuvant chemotherapy and trastuzumab were included. Table 1 showed clinicopathological characteristics of the training set (101 patients), internal testing set (57 patients), and external independent testing sets (53 patients). HR positive tumor comprised 50.5%, 65.0%, and 68% of patients in each cohort, respectively. The median follow-up time was 58.4 months (IQR 42.8–76.9), and 49 of 211 patients (23.2%) developed tumor relapse during the follow-up period.

Firstly, we compared the global and targeted miRNA expression profiling in another 14 FFPE primary breast tumor specimens, including seven non-recurrent cases (group A) and seven recurrent cases (group B). There was no statistically difference between the two groups in terms of clinical characteristics, except the DFS (Supplementary Table 1). The median DFS was 81.5 ± 37.0 and 24.2 ± 7.1 months in group A and group B, respectively. As a result, nine miRNAs that were significantly differentially expressed between the two groups were identified. (Table 2; Supplementary Figure 1)

Then, the expression of the nine miRNAs was confirmed using qRT-PCR analysis in the training set. The optimum cutoff values for these candidate miRNAs were generated by X-tile plots, which translate miRNA data from continuous variable into categorical variable (high expression or low expression). (Supplementary Figure 2A–I)

After that, we put each miRNA status (high or low expression), together with DFS data, into COX regression formula, and thus identified 2 miRNA (mir-150-5p and mir-4734) that were independently significantly associated with DFS. (Table 3) Next, a formula based on the two miRNA status and their impact on DFS (represented by theβcoefficient in COX regression model) was generated to calculate the risk score for the hazard of disease recurrence. The risk score = (1.642* status of miR-150-5p)−(1.721* status of miR-4734), where low expression status equals 0 and high expression status equals 1.

The risk score of each patient in the training set was calculated, and then took into X-tile plot. As a result, −0.1 was selected as the optimal cut-off value of risk score. (Supplementary Figure 2J) Therefore we classified those patients with risk score ≥ −0.1 as high-risk group, and those with risk score < −0.1 as low-risk group.

We further compared the prognostic predict performance of 9-miRNA model with 2-miRNA model in the training set using time-ROC curve. As a result, 2-miRNA set was significantly superior to the 9-miRNA set (p = 0.034) (Supplementary Figure 3A) Besides, the kaplan-meier curve also suggested 2-miRNA set had an advantage over 9-miRNA set. (Supplementary Figure 3B), which supporting the establishment of 2-miRNA signature.

### Validation of the miRNA prognostic classifier

**Figure 1:**
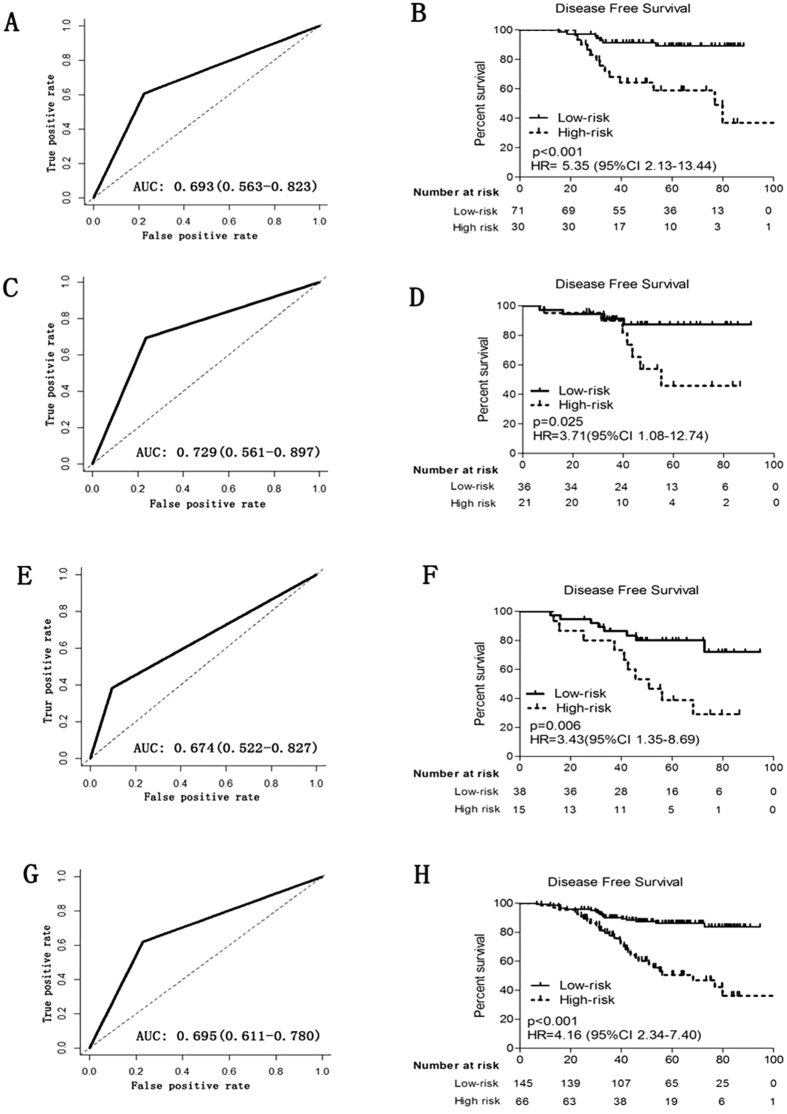
Stratified by the 2 miRNA-based classifier, time-dependent ROC curves and Kaplan-Meier survival of the training, internal testing, external independent sets and combination set. Data are AUC (95% CI) or hazard ratio (95% CI). ROC = receiver operator characteristic. AUC = area under the curve. Training cohort.(**A**,**B**) internal testing cohort. (**C**,**D**), external independent validation cohort (**E**,**F**) and combination set(**G**,**H**). We used AUCs at 5 years to assess prognostic accuracy, and calculated p values using the log-rank test.

Patients in the low-risk group generally had better disease free survival than those in the high-risk group. There was no significant difference in the distribution of clinicopathological features between the high-risk and low-risk group in each set (Table 1). In the training set, 5-year disease-free survival was 59.0% for the high-risk group and 89.2% for the low-risk group (hazard ratio [HR] 5.35, 95% CI 2.13–13.44; p < 0.001) (Fig. 1B).

We did the same analysis using in the internal testing cohort. Five-year disease-free survival was 45.8% for the high-risk group and 87.5% for the low-risk group (HR 3·71, 95% CI 1.08–12.74; p = 0.025). (Fig. 1D)

To confirm that 2-miRNA based classifier had consistent prognostic value in different populations, we applied it to the external independent testing set. Five-year disease-free survival rate was 38.9% for the high-risk group and 80.1% for the low-risk group (HR 3·43, 95% CI 1.35–8.69; p = 0.006) (Fig. 1F).

Univariate analysis showed that 2-miRNA signature was significantly associated with DFS in each cohort. (Supplement Table 2) After multivariable adjustment by other prognostic factors including HR status, TNM stage, tumor grade and age, the 2-miRNA based model remained a powerful and independent factor in the entire cohort of 211 cases (HR 4.63, 95% CI 2.45–8.74, p < 0.0001) (Table 4).

**Figure 2:**
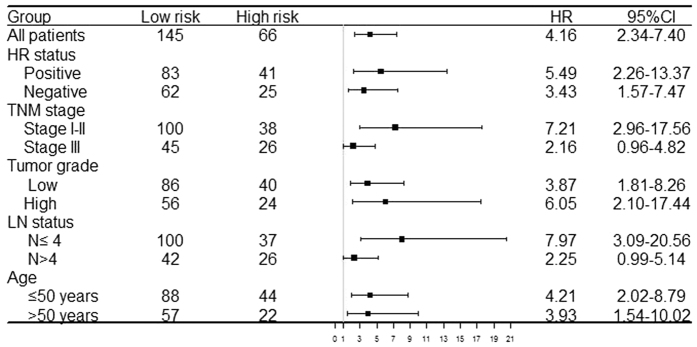
Effect of 2-miRNA based signature on DFS in different subgroups.

When stratified by clinicopathological risk factors, the 2-miRNA based classifier still showed clinically and statistically significant prognostic effect in all subgroups (Fig. 2).

### Comparing the prognostic performance of miRNA classifier with other clinicopathological factors

To further evaluate the prognostic performance of the miRNA signature, we assessed the prognostic accuracy of the 2-miRNA based classifier with time-dependent ROC analysis at five years, and calculated the AUC of the ROC curves for disease recurrence in all 211 patients.

**Figure 3:**
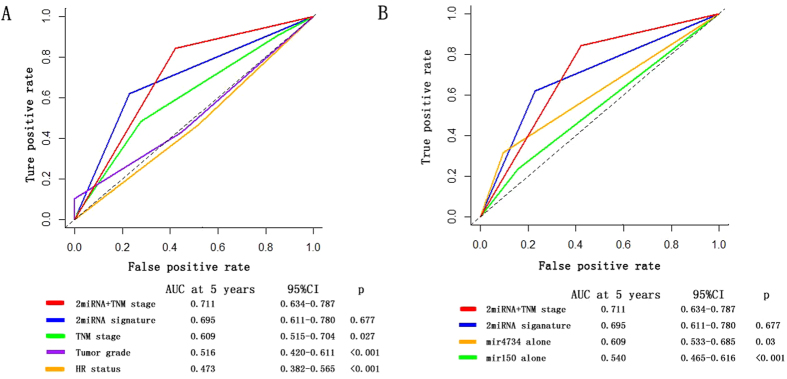
Time-dependent ROC curves compare the prognostic accuracy of the 2-miRNA based signature with clinicopathological risk factors and single miRNAs in all 211 patients with HER2 positive breast cancer. ROC = receiver operator characteristic. AUC = area under curve. HR = hormonal receptor. (**A**) Comparisons of the prognostic accuracy by the two-miRNA-based signature (high risk vs low risk), TNM stage (Stage1 vs Stage2 vs stage 3), pathological tumor grade (high vs low), and the miRNA signature and TNM stage combined. (**B**) Comparisons of the prognostic accuracy by the two-miRNA-based signature (high vs low risk), the miRNA signature and TNM stage combined, the miR-4734 alone (high vs low expression) and miR-150-5p alone (high vs low expression).

The 2-miRNA signature showed an AUC of 0.693 (95% CI 0.563–0.823), 0.729 (95% CI 0.561–0.897), and 0.674 (95% CI 0.522–0.827) in the training, internal and external independent set, respectively (Fig. 2A,C,E). In the whole cohort of 211 patients, the 2-miRNA based classifier showed higher prognostic accuracy than other clinicopathological risk factor, including TNM stage, HR status, tumor grade and age (Fig. 3).

Eventually, the combination of TNM stage and 2-miRNA based classifier demonstrated the highest prognostic accuracy than any other prognostic factors or miRNA alone, with an AUC of 0.711 (95% CI 0.634–0.787). In addition, the performance of 2miRNA+TNM stage was statistically superior to TNM stage alone (AUC: 0.711 vs.0.609, p = 0.027), supporting the addition of 2miRNA to traditional TNM stage as a prognostic factor (Fig. 3).

Collectively, our results demonstrate that expression of a small set of miRNA, measured from primary breast cancer tissues at initial diagnosis, was a valid prognostic indicator and will improve the prognostic capacity of AJCC stage for the development of disease recurrence.

## Discussion

In this study, we developed and confirmed, for the first time, a novel prognostic model based on 2-miRNA expression to improve the prediction of disease recurrence in patients with HER2 positive breast cancer who completed standard treatment. Our results clearly demonstrated that this classifier can successfully stratified patients into two groups by their risk of tumor recurrence, regardless of the clinical features. Furthermore, this signature predicted the five year DFS better than other clinicopathological factors, and added prognostic value to the TNM staging system.

The value of miRNA as prognostic biomarkers has been increasingly explored^18^,^19^,^20^. However, it have not been comprehensively studied in patients with primary HER2 positive breast cancer. Jung *et al*.^14^ showed that circulating miR-210 levels were associated with trastuzumab sensitivity, tumor presence, and lymph node metastases in patients who received neo-adjuvant trastuzumab based chemotherapy. In contrast to short-term efficacy, the present study focused on the association between miRNA and long-term benefit of trastuzumab based treatment. We accessed to large numbers of primary tumor tissues with extensive clinical follow-up in distinct study cohorts to confirm the robustness of this miRNA based signature as a useful predictor of long-term prognosis.

Our results suggested that incorporation of miRNA signature into the conventional clinical factors can provide more accurate prognostic information. The miRNA signature successfully distinguished patients with similar clinical features into distinct groups depending on their risk of tumor recurrence. Besides, combination of miRNA signature and TNM system improved the prognostic predict performance than other models including miRNA alone or TNM stage alone. Therefore, the novel miRNA classifier is valuable, in supplement of current TNM system, to define more accurate prognosis, and had the potential to improve the management of HER2 positive breast cancer patient.

Our data suggested that patients with high risk of recurrence may be inadequately treated with the currently available treatment. Presently, strategies of escalating adjuvant anti-HER2 treatment have been pursued. Neratinib significantly reduced the risk of recurrence by 33% versus placebo at first two years, in patients who have completed the chemotherapy and one year of trastuzumab^21^. The effect of combination of trastuzumab and pertuzumab in adjuvant setting was also examined in phase III APHINITY trial. Therefore, development of this miRNA based prognostic assay will contribute to identify patients who may benefit most from more extensive adjuvant therapies, and thus help to tailor the adjuvant anti-HER2 treatment.

The biologic function of the two miRNAs in breast cancer still needs to be established. Increased expression of mir150 was reported to correlate with poorer clinical outcome in intrahepatic cholangiocarcinoma^22^ and non-small cell lung cancer^23^. Besides, recent evidence showed that mir150 was member of a miRNA based prognostic model for primary melanoma, and was associated with CD45+ TILs in tumor tissues^18^. On the other hand, mir4734 is a newly identified miRNA in breast cancer by extensive next-generation sequencing analysis. It encodes within the ERBB2/Her2 gene, which is amplified in HER2 positive breast cancer and cause the clinically genomic aberration^24^. Our work provided new evidence revealing the association between mir4734 expression and clinical outcome of HER2 positive breast cancer, which may aid further exploration of potent biological function.

The major limitation of this work is the small sample size of the original cohort selected for microarray study. Be aware of this issue, several efforts were made to make up for the deficiency. Firstly, the original groups of patients for miRNA screening were carefully selected, matching all the clinical prognostic factors well, and making DFS being the only significantly different factor. Secondly, the preliminary result of microarray experiment was strictly validated with larger sample size using appropriate methods. Besides, a large, multicenter prospective study to assess the robustness of prognostic signature in the general HER2 positive breast cancer population is required.

In summary, the described miRNA signature represented the first step to develop a molecular prognostic assay for HER2 positive breast cancer. We believe such a model has the potential to improve the management of this specific population.

## Methods

### Study population

We used formalin-fixed paraffin-embedded (FFPE) tissue samples from 211 patients with stage I-III HER2 positive breast cancer. For the training and internal testing set, data were obtained from 158 patients in Cancer Hospital, Chinese Academy of Medical Sciences, Beijing, China, between June 1, 2000, and June 30, 2015. We used computer-generated random numbers to assign 101 of these patients to the training set, and 57 patients to the internal testing set. We enrolled another 53 patients, with the same criteria as above, from other 10 hospitals in China as the independent validation set.

HER2-positive was defined as 3+ on immunohistochemical [IHC] analysis or 2+ with gene amplification by fluorescence *in situ* hybridization [FISH]. Informed consent was obtained from all patients and approval acquired by the Institutional Review Board. Clinical information relevant to this study include date of diagnosis, date of recurrence or last follow-up or death, age, HR status, tumor grade, TNM stage for primary tumors and menopausal status. We defined disease free survival as the time from the date of surgery to the date of confirmed tumor relapse or the date of last follow-up visit for disease-free patients. We excluded patients who had no FFPE tumor sample from initial diagnosis, or insufficient RNA (less than 100 ng/μL) available. All the patients provided informed consent. The study was performed in accordance with Declaration of Helsinki and approved by ethics committee in Cancer Hospital, Chinese Academy of Medical Sciences.

### Clinical specimens

All specimens were human primary breast cancer samples that were collected, formalin-fixed, and paraffin-embedded at the time of surgery. All tumors were classified according to the 2010 American Joint Committee on Cancer (AJCC) staging system

### RNA extraction

All the FFPE tissues comprised at least 80% tumor cells. RNA extraction was performed using the miRNeasy FFPE Kit (Qiagen) following manufacturer’s recommendations, using the Xylene/Ethanol method for deparaffinization/rehydration.

### microRNA microarray expression profiling and data preprocessing

To generate miRNA expression profiles, we selected another panel of FFPE tumor samples from 14 patients including seven relapsed disease and seven non-relapsed. The miRNA profiling was performed using Agilent miRNA array, which contained probes interrogating 2006 human mature miRNAs from miRBase R19.0. Microarray experiments were conducted according to the manufacturer’s instructions. Briefly, the miRNAs were labeled using the Agilent miRNA labeling reagent. Total RNA (100 ng) was dephosphorylated and ligated with pCp-Cy3, the labeled RNA was purified and hybridized to miRNA arrays. Images were scanned with the Agilent microarray scanner (Agilent), gridded, and analyzed using Agilent feature extraction software version 10.10. The miRNA array data were analyzed for data summarization, normalization and quality control by using the GeneSpring software V12 (Agilent). To select the differentially expressed genes, we used threshold values of 2-fold change and a Benjamini-Hochberg corrected p vlaue of 0.05. The data was Log2 transformed and median centered by genes using the Adjust Data function of CLUSTER 3.0 software then further analyzed with hierarchical clustering with average linkage. Finally, we performed tree visualization by using Java Treeview (Stanford University School of Medicine, Stanford, CA, USA).

### microRNA real-time qPCR and data processing

On the basis of the miRNA microarray results, we further examined miRNA expression using qRT-PCR to analyze the 211 FFPE samples to validate the prognostic value of every candidate miRNA. One microgramme of RNA was reverse-transcribed in 25-mL reactions using the Superscript II reverse transcriptase (Invitrogen) according to the manufacturer’s instructions. Quantitative real-time PCR (qPCR) was conducted using the SYBR Premix Ex TaqTM II (TliRNaseH Plus) kit (TaKaRa, Japan) with the Bio-Rad (USA) machine. U6 small nuclear RNA was used as internal normalized references. Expression levels of individual miRNA were determined by −ΔCT approach (ΔCT = CT miRNA − CT U6 RNA).

### Statistical analysis

We compared two groups using χ^2^ test for categorical variables. For survival analyses, we used the Kaplan-Meier method to analyze the correlation between variables and disease-free survival, and the log-rank test to compare survival curves. We used the Cox regression model to do the multivariable survival analysis. Statistical significance was set at 0.05. All these statistical tests were done with SPSS version 17.0 (SPSS Inc., Chicago. USA).

We selected the optimum cutoff score for the expression of every miRNA using X-tile plots (version 3.6.1 (Yale University School of Medicine, New Haven, CT, USA) based on the association with the patients’ disease-free survival. X-tile plots provide a single and intuitive method to assess the association between variables and survival^25^.

We investigated the prognostic accuracy of each feature and multi-miRNA based classifier using time-dependent receiver operating characteristic (ROC) analysis^26^. We used the area under the curve at 5 years to measure prognostic accuracy. We used R software version 3.1.3 and the “time ROC” package to do the time-dependent ROC curve analysis.

## Additional Information

**How to cite this article**: Du, F. *et al*. A miRNA-based signature predicts development of disease recurrence in HER2 positive breast cancer after adjuvant trastuzumab-based treatment. *Sci. Rep.* **6**, 33825; doi: 10.1038/srep33825 (2016).

## Supplementary Material

Supplementary Information

## Figures and Tables

**Table 1:** Baseline characteristics of patients by miRNA assessment set.

	Training set (n = 101)			Internal testing set (n = 57)			External Independent set (n = 53)		
	Number of patients	Low risk (n)	High risk (n)	Number of patients	Low risk (n)	High risk (n)	Number of patients	Low risk (n)	High risk (n)
Age									
≤50	58	41	17	33	18	15	41	29	12
>50	43	30	13	24	18	6	12	9	3
TNM stage									
I	21	16	5	2	1	1	1	0	1
II	45	34	11	33	25	8	36	24	12
III	34	21	13	21	10	11	16	14	2
Unknown	1	0	1	1	1	0	0	0	0
Tumor grade									
Good	60	41	19	37	22	15	29	23	6
Poor	39	28	11	19	14	5	22	14	8
Unknown	2	2	0	1	0	1	2	1	1
HR status									
Positive	51	38	13	39	23	16	34	22	12
Negative	50	33	17	18	13	5	19	16	3
Chemotherapy									
EPI-included	69	51	18	41	25	16	37	27	10
Non-EPI	31	19	12	14	9	5	14	10	4
Unknown	1	1	0	2	2	0	2	1	1

**Table 2:** miRNA expression profiles in 7 relapsed vs.7non-relapsed tumor tissues of HER2 positive breast cancer.

miRNA	Relapsed tumor	Non-relapsed tumor	Fold change	p value
	mean expression	mean expression		
Higher expression in relapsed tumor				
hsa-miR-361-5p	26.9	12.2	14.3	0.025
hsa-miR-26a-5p	309.4	174.5	2.0	0.027
hsa-miR-365a-3p	65.6	24.4	21.1	0.028
hsa-miR-155-5p	45.9	21.4	15.6	0.034
hsa-miR-205-5p	484.7	232.2	2.4	0.038
hsa-miR-150-5p	203.2	107.3	2.2	0.042
hsa-miR-106b-5p	66.0	43.4	17.3	0.043
Lower expression in relapsed tumor				
hsa-miR-4734	20.9	47.9	12.3	0.039
hsa-miR-424-3p	6.9	18.1	16.1	0.034

**Table 3:** Members of the 2-miRNA signature predicting disease recurrence of HER2 positive breast cancer derived from Cox proportional hazards modeling in the training set.

Parameters	β	SE	Wald	df	p	Hazard Ratio^a^	95% CI
**mir150**	1.642	0.537	9.363	1	0.002	5.17	1.81–14.79
**mir4734**	−1.721	0.564	9.321	1	0.002	0.18	0.06–0.54

**Table 4:** Multivariable Cox regression analysis of two miRNA based signature, clinicopathological characteristics with disease-free survival.

Variables	Training cohort (n = 101)		Combined internal and external cohort (n = 110)		Entire cohort (n = 211)	
	p value	HR(95% CI)	p value	HR(95% CI)	p value	HR(95% CI)
Age(≤50 years vs.>50 years)	0.311	1.67(0.62–4.49)	0.309	1.59(0.65–3.86)	0.306	1.39(0.74–2.612)
TNM stage (Stage1 vs.2)	0.150	4.92(0.56–43.02)	0.008	0.13(0.03–0.59)	0.826	1.13(0.38–3.42)
TNM stage (Stage1 vs.3)	0.129	5.02(0.63–40.26)	0.180	0.35(0.07–1.63)	0.173	2.12(0.72–6.25)
Tumor grade(Poor vs.Good)	0.482	1.45(0.51–4.13)	0.691	1.20(0.49–2.94)	0.933	1.03(0.55–1.93)
HR status(Negative vs.Positive)	0.085	0.36(0.11–1.15)	0.991	1.01(0.42–2.40)	0.279	0.71(0.38–1.33)
2mirRNA signature(High. vs.Low)	<0.001	7.07(2.40–20.79)	0.007	3.19(1.38–7.39)	<0.001	4.63(2.45–8.74)
