# Erratum: A miRNA-based signature predicts development of disease recurrence in HER2 positive breast cancer after adjuvant trastuzumab-based treatment

**DOI:** 10.1038/srep35509

**Published:** 2016-10-14

**Authors:** F. Du, P. Yuan, Z. T. Zhao, Z. Yang, T. Wang, J. D. Zhao, Y. Luo, F. Ma, J. Y. Wang, Y. Fan, R. G. Cai, P. Zhang, Q. Li, Y. M. Song, B. H. Xu

Scientific Reports 6: Article number: 3382510.1038/srep33825; published online: 09 21 2016; updated: 10 14 2016

The Acknowledgements section in the PDF version of this Article is incorrect.

“The following institutions participated in this study: Fudan University Shanghai Cancer Center; Zhejiang Cancer Hospital; Guangdong General Hospital; Tongji Hospital, Tongji Medical College Huazhong University of Science and Technology; Nanfang Hospital, Southern Medical University; Sun Yat-Sen University Cancer Hospital; West China Hospital, Sichuan University; Harbin Medical University Cancer Hospital; Henan Cancer Hospital, Zhengzhou University; Peking Union Medical College Hospital. This research was supported by a grant of the Korea Health Technology R&D Project through the Korea Health Industry Development Institute (KHIDI), funded by the Ministry of Health & Welfare, Republic of Korea (HI14C0466), and funded by the Ministry of Health & Welfare, Republic of Korea (HI14C3344)”.

should read:

“The following institutions participated in this study: Fudan University Shanghai Cancer Center; Zhejiang Cancer Hospital; Guangdong General Hospital; Tongji Hospital, Tongji Medical College Huazhong University of Science and Technology; Nanfang Hospital, Southern Medical University; Sun Yat-Sen University Cancer Hospital; West China Hospital, Sichuan University; Harbin Medical University Cancer Hospital; Henan Cancer Hospital, Zhengzhou University; Peking Union Medical College Hospital. This work was supported by Beijing Hope Run Special Fund (LC2013L09) and Capital Clinical Feature Applied Research Fund (Z141107002514010). The funders had no role in study design, data collection and analysis, decision to publish, or preparation of the manuscript”.

