# Supplementary material for: A miRNA-based signature predicts development of disease recurrence in HER2 positive breast cancer after adjuvant trastuzumab-based treatment
Source: Sci Rep. 2016 Sep 21;6:33825. doi: 10.1038/srep33825 (PMC5030658; doi:10.1038/srep33825)
Supplement: Supplementary Information [file srep33825-s1.pdf]

A miRNA-based signature predicts development of disease recurrence in HER2 positive breast cancer after adjuvant trastuzumab-based treatment

F Du[1,2*], P Yuan[1*], Z.T Zhao[3], Z Yang[4], T Wang[5], J.D Zhao[1], Y Luo[1], F Ma[1], J.Y Wang[1], Y Fan[1], R.G Cai[1], P Zhang[1], Q Li[1], Y.M Song[3], B.H Xu[1]

*F Du and P Yuan contributed equally to this work

1 Department of Medical Oncology, Cancer Hospital, Peking Union Medical College and Chinese Academy of Medical Sciences, Beijing, China

2 The VIPII Gastrointestinal Cancer Division of Medical Department, Peking University Cancer Hospital and Institute, 52 Fucheng Road, Haidian District, Beijing, China

3 State Key Laboratory of Molecular Oncology, Cancer Hospital, Chinese Academy of Medical Sciences and Peking Union Medical College, Beijing, China

4 Department of Cancer Epidemiology, Cancer Hospital, Chinese Academy of Medical Sciences and Peking Union Medical College, Beijing, China

5 Tumor Marker Research Center, Cancer Institute and Hospital, Chinese Academy of Medical Sciences and Peking Union Medical College, Beijing, China

Supplementary Figure 1

Hierarchical clustering of seven tumor recurrence cases versus seven non-relapsed tumor tissues with the nine differentially expressed miRNAs using Euclidean distance and average linkage clustering. Every row represents an individual gene, and each column represents an individual sample.

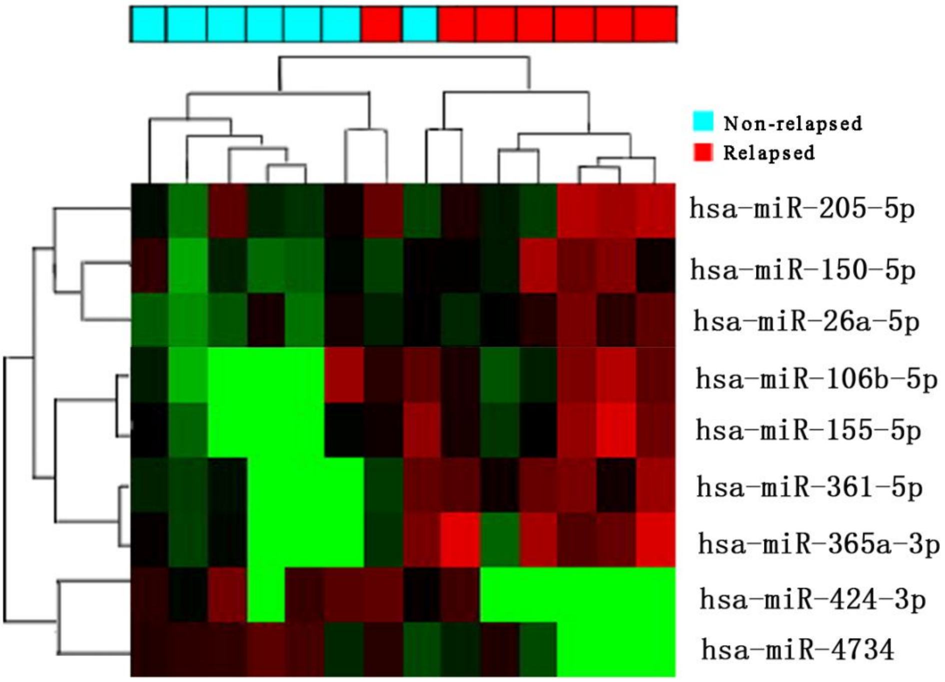

Supplementary Figure 2

X-tile plots of the nine candidate miRNAs and 2miRNA risk value in the training set

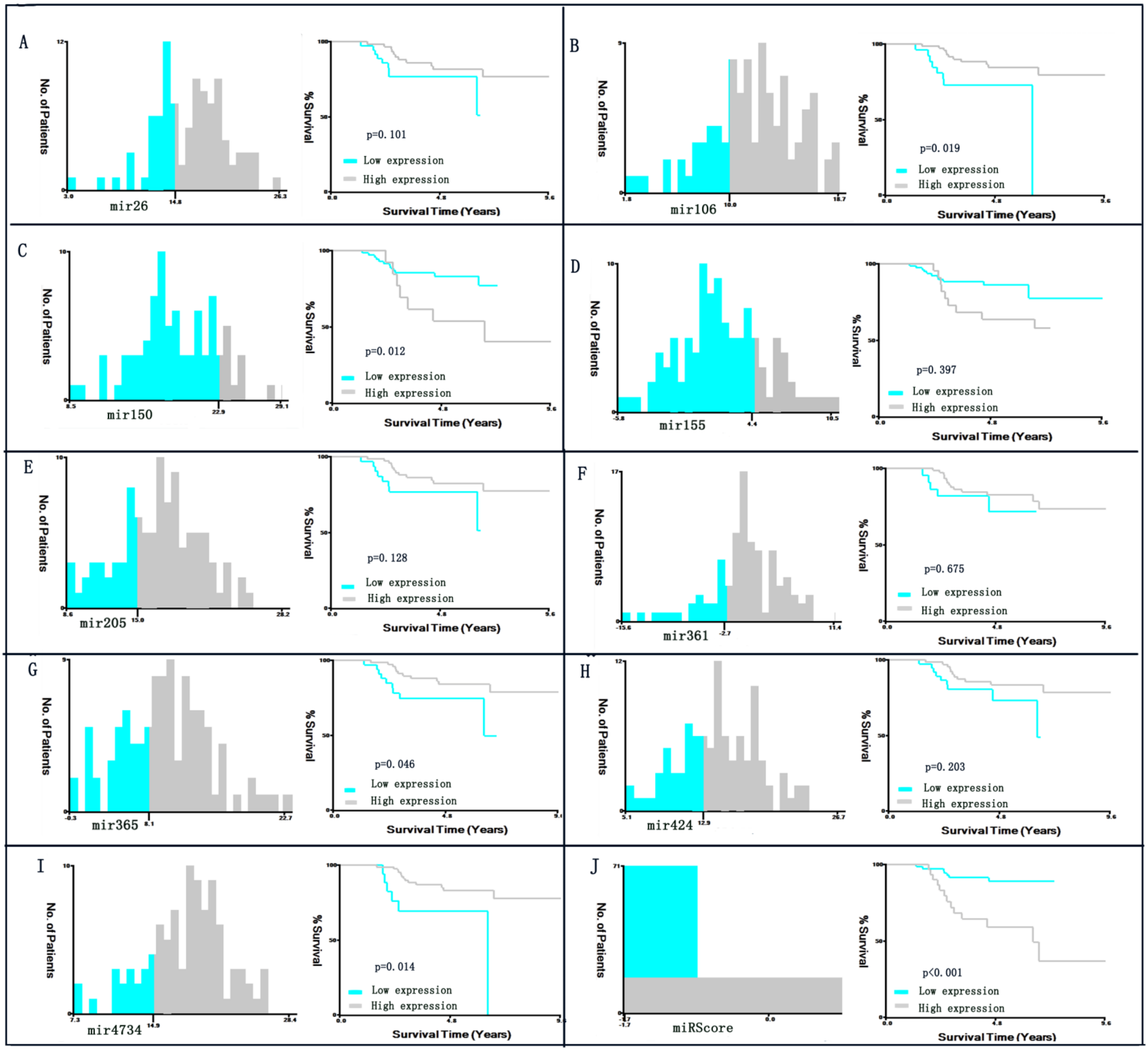

Supplementary Figure 3

(A) Time-dependent ROC curves compare the prognostic accuracy of the 2-miRNA signature with 9-miRNA signature in training set.
ROC=receiver operator characteristic.
AUC=area under curve.HR=hormonal receptor.

(B) Kaplan-Meier curve of the low-risk group base on 9-miRNA signature, stratified by the 2-miRNA signature.

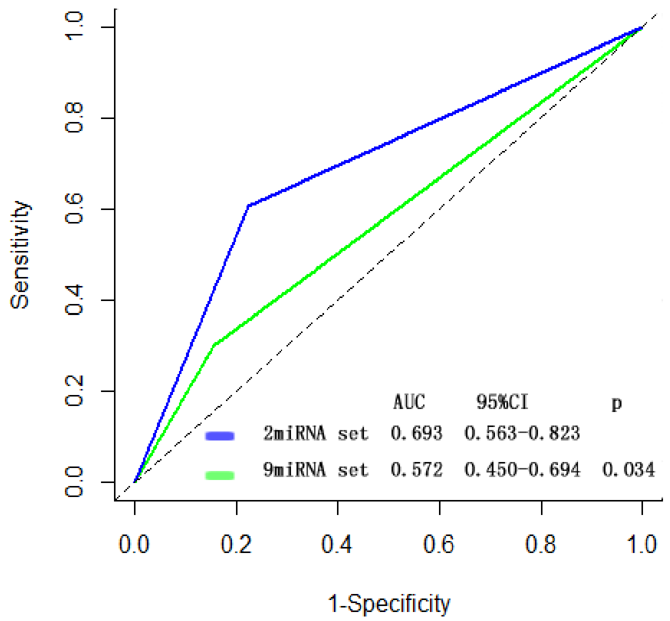

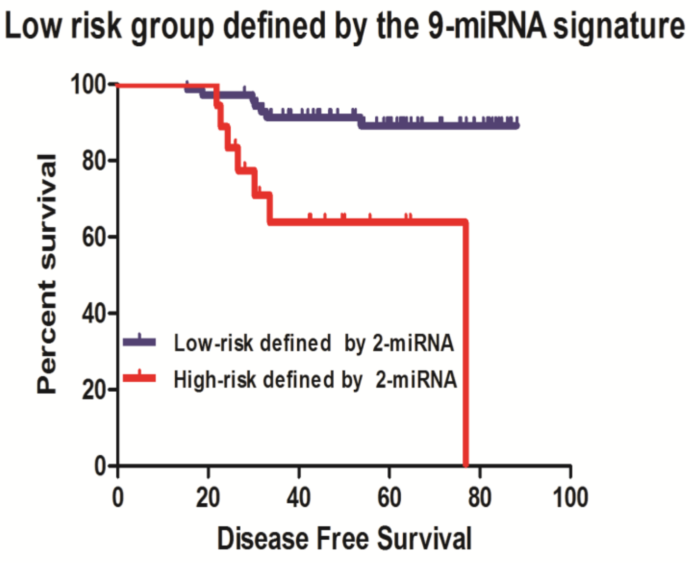

Supplementary Table 1

Clinical characteristics of the 7 non-recurrent patients (Group A) and 7 recurrent patients (Group B)

Patient Code	Recurrence status	T stage	N stage	HR status	Tumor Grade	Radio-therapy	DFS (Month)	Metastatic sites
A1	0	2	1	P	2	Yes	44.4	
A2	0	1	1	N	2	No	144.0	
A3	0	2	1	N	2	Yes	68.0	
A4	0	1	1	N	2	No	120.0	
A5	0	1	2	P	2	Yes	49.4	
A6	0	2	1	N	2	Yes	65.7	
A7	0	2	0	P	3	No	78.9	
B1	1	1	2	P	3	Yes	19.5	Liver
B2	1	1	2	P	2	Yes	25.6	Lung
B3	1	2	2	N	2	Yes	31.7	Liver
B4	1	2	1	N	3	No	17.1	Contralateral breast
B5	1	2	1	N	2	Yes	21.6	Lymph nodes
B6	1	2	1	P	3	Yes	18.4	Liver
B7	1	1	1	P	3	Yes	35.8	Bone

Abbreviation:
HR: hormone receptor
P: positive; N: negative

Supplement Table.2

Univariate association of two-miRNA-based signature, clinicopathological characteristics, and single miRNAs with disease-free survival

Variations	Training set (n=101)		Internal testing set (n=57)		External Independent set (n=53)	
	HR(95%CI)	p	HR(95%CI)	p	HR(95%CI)	p
**Age(≤50 years vs.>50 years)**	0.89(0.37-2.17)	0.804	1.25(0.37-4.28)	0.719	0.60(0.21-1.67)	0.320
**TNM stage (Stage1+2 vs.3)**	1.75(1.11-2.76)	0.011	1.93(1.02-3.65)	0.032	1.25(0.78-2.01)	0.355
**Tumor grade(High vs.Low)**	1.16(0.71-1.89)	0.556	1.17(0.60-2.31)	0.646	1.04(0.63-1.70)	0.882
**HR status (Neg vs.Pos)**	1.60(0.99-2.59)	0.045	1.50(0.83-2.72)	0.169	0.94(0.58-1.54)	0.816
**mir4734 alone**	0.30(0.11-0.78)	0.009	0.27(0.06-1.11)	0.052	0.22(0.09-0.55)	<0.001
**mir150 alone**	3.25(1.29-8.20)	0.008	2.02(0.59-6.93)	0.255	2.89(0.37-22.77)	0.314
**2mirRNA signature**	5.35(2.13-13.44)	<0.001	3.71(1.08-12.74)	0.025	3.43(1.35-8.69)	0.006
